# Impact of the COVID-19 Pandemic on the Public Perceptions of the Roles and Functions of Community Pharmacies in South Korea: Updated Cross-Sectional Self-Reported Web-Based Survey

**DOI:** 10.2196/46723

**Published:** 2023-07-13

**Authors:** Dong-Wook Yang, Kyung-Bok Son

**Affiliations:** 1 School of Pharmacy Sungkyunkwan University Suwon, Gyeonggi-do Republic of Korea; 2 College of Pharmacy Hanyang University Ansan, Gyeonggi-do Republic of Korea

**Keywords:** community pharmacy, pharmaceutical practice, COVID-19, South Korea, pandemic, survey, pharmacists, pharmacy, primary care, medicine-centered services

## Abstract

**Background:**

Community pharmacists confronted dual burdens in response to the COVID-19 pandemic by expanding the scope of pharmaceutical practices.

**Objective:**

This study aimed to assess the perceived roles and functions of community pharmacies during the pandemic and to explore their updated roles after the pandemic began.

**Methods:**

We conducted a self-reported web-based survey in October 2022. Based on Korean census data, we recruited the study participants (n=1000) through quota sampling stratified by age, sex, and region, yielding a 7.45% (1000/13,423) response rate. The questionnaires were composed of 3 sections: demographics, the roles and functions of community pharmacies during the pandemic, and the updated roles of community pharmacies during disasters. Each question in the second and third sections was rated on a 5-point Likert scale from 1 (strongly disagree) to 5 (strongly agree), and each item’s mean scores and SDs were reported. The study participants were categorized into 2 groups: individuals who had a family pharmacy and those who did not. A chi-square test and ordered logistic regression analyses were conducted.

**Results:**

Out of 1000 respondents, 418 (41.8%) had a history of COVID-19, and 639 (63.9%) had a family pharmacy. Assigning specific roles and functions to community pharmacies during the pandemic contributed to positive assessments. Respondents gave higher scores to community pharmacies that had responded appropriately (a mean Likert score of 3.66, SD .077 out of 5) and provided continuous pharmaceutical services (mean 3.67, SD 0.87) during the pandemic. The pandemic served as an opportunity to positively recognize the role of community pharmacies (mean 3.59, SD 0.83). In the ordered logistic model, having a family pharmacy was consistently associated with positive perceptions. Respondents perceived that community pharmacies collaborated with general practitioners and health authorities. However, community pharmacies need to function appropriately in terms of knowledge. The mean score of the 4 domains of community pharmacy functions was the highest for collaboration (mean 3.66, SD 0.83), followed by communication (mean 3.57, SD 0.87), responsiveness (mean 3.54, SD 0.87), and knowledge (mean 3.41, SD 0.91).

**Conclusions:**

The pandemic resulted in interprofessional collaboration between community pharmacists and general practitioners. Family pharmacies could be a valuable asset to the comprehensive case management of patients. However, community pharmacists should have the expertise to build solid interprofessional collaborations and fulfill their expanded and updated roles.

## Introduction

### Background

Pharmacists are the third-largest group of health care professionals after nurses and physicians [1,2]. The most common type of pharmacy is a community pharmacy [3]. Community pharmacies include retail pharmacies that provide over-the-counter drugs and other health-related products to a specific community [4] and outpatient pharmacies that are considered an essential part of the primary care system [5]. Community pharmacists are the most accessible and visited health care professionals, and community pharmacies are the public’s first access to their medication. Given their roles and functions, community pharmacists are integral health care professionals. However, community pharmacies are not integrated into the current primary care system [6,7]. Community pharmacists operate in a retail environment, selling or dispensing medications independently [8], and remain underestimated in their roles and functions as an integral part of the health care system [9].

In an aging population, community pharmacies are increasingly required to expand the scope of traditional pharmaceutical practices [10-12]. The health care demands of patients exceed physicians’ capacity to address them [13]. Pharmaceutical practices can be categorized into medicine-centered services, patient-centered care, and public health services [14-16]. Medicine-centered services indicate traditional pharmaceutical care, such as promoting the safe use of medicines, providing information on medicines, and dispensing prescriptions. Patient-centered care emerged as an essential element of high-quality health care [17], and patient-centered pharmaceutical care includes counseling services and managing specific disease states [18-20]. Public health services may include pharmacovigilance, monitoring infectious diseases, and education in disease prevention [15,21]. Several policies have been implemented to expand the scope of pharmaceutical practice care from medicine-centered services to patient-centered care and public health services [12-15].

Community pharmacies are currently compelled to be on the frontline against COVID-19 [22,23], and the expanded role of community pharmacies has gained momentum within a short time frame [24]. As the spread of the COVID-19 pandemic continues, unprecedented measures, such as social distancing restrictions, mandated stay-at-home rules, and business closures, have been implemented to contain the transmission of the virus, control people who have been infected, and prevent overwhelming the health system [25-28]. The increased number of people with COVID-19 has caused a surge in the demand for health care services [29-31]. Further, the need for pharmaceutical services during the pandemic is beyond the scope of pharmaceutical services in the nonpandemic situation. According to the 4 disaster phases, the updated need for pharmaceutical services could be divided into prevention, preparedness, response, and recovery [32,33]. The public needs to be informed about adequate personal protection against the infection and community transmission of COVID-19. Patients must be treated for the disease and other related symptoms at home with rational medication use.

### Pharmaceutical Practice in South Korea

Pharmacy practice in South Korea needs to catch up to practices in other countries. The concept of pharmaceutical care was first introduced in 1994 [34]. However, pharmacy practices are still limited to medicine-centered services. Pharmacists in South Korea do not have the authority to direct refill reviews and approvals and administer vaccinations [11]. South Korea introduced a law separating prescribing and dispensing medicine in 2000 [35]. Under the law, only physicians could prescribe medicines, and pharmacists could dispense the medicine by prescription. The law aimed to enhance the rational use of medicine and manage pharmaceutical expenditures [36]. However, the separate systems relegated community pharmacies to delivering medicine-centered services, only dispensing medicines rather than performing other pharmacy practices [11]. Thus, community pharmacies are located near clinics or hospitals where they receive and dispense prescriptions [37]. Patients visit community pharmacies near clinics or hospitals to fill prescriptions; they usually receive prescriptions when they visit clinics or hospitals and then fill those prescriptions at different community pharmacies. Thus, providing centralized and consistent pharmaceutical practices is difficult in South Korea. The Korean Pharmaceutical Association has suggested a family pharmacy system to the government to provide effective and comprehensive pharmaceutical services [37]. However, a family pharmacy is still not a legally established concept in the Korean health care system.

In South Korea, the COVID-19 pandemic has changed the roles and functions of community pharmacies in 2 ways. The expanded role of traditional community pharmacies requested by the community has been accelerating quickly. Furthermore, community pharmacies must develop new functions to address the pandemic, particularly in their preparedness and responses to COVID-19. We considered the interaction between the 2 burdens—increased and updated demand for pharmaceutical practice—placed on the current community pharmacies. The primary objective of this study was to assess the perceived roles and functions of community pharmacies during the pandemic and explore their updated roles, not to test a hypothesis but to measure the public perceptions toward community pharmacies. The secondary objective was to investigate the role of family pharmacies in the public perceptions of community pharmacies.

## Methods

### Overview

We conducted this study as part of a more extensive study on the public perceptions of the roles and functions of community pharmacies in South Korea. Public perceptions before the COVID-19 outbreak have been published elsewhere [11]. This study investigated the changes in the public perceptions of community pharmacies during the pandemic. To this end, we developed a self-reported web-based survey. The survey was conducted from October 2 to October 4, 2022, with technical support from Realmeter, a survey agency. The characteristics of the previous and current studies are provided in Multimedia Appendix 1.

### Study Design and Participant Recruitment

Assuming a 95% CI, 3.1% margin of error, and 0.5% SD, a representative sample size (n=1000) for the entire population was obtained. A quota sampling method was implemented to recruit study participants. The participants were registered in the survey agency’s panel and agreed to respond to the survey in advance. Quotas for the sample were stratified by sex, age, and region based on Korean census data. The study population was Korean individuals aged 19 years or older. For the quota sampling process, regions were categorized into *Si* and *Do*. *Si* indicates an urban area in South Korea and includes 8 regions: Seoul, Busan, Daegu, Incheon, Kwangju, Daejeon, Ulsan, and Sejong. Do indicates a rural area in South Korea and includes 9 regions: Gyeonggi, Gangwon, Chungbuk, Chungnam, Jeonbuk, Jeonnam, Gyeongbuk, Gyeongnam, and Jeju. *Do* indicates a rural area in Korean and includes 9 regions: Gyeonggi, Gangwon, Chungbuk, Chungnam, Jeonbuk, Jeonnam, Gyeongbuk, Gyeongnam, and Jeju. Invitations were sent randomly for the stratified quotas until the number of completed surveys reached the predetermined percentage.

The survey was conducted as follows. We explained the purpose of the study. Study participants who consented to participate in this study were directed to the encrypted website to complete the survey. Those who completed the survey received a voucher worth US $4. Out of 13,423 invitations, 1000 completed the survey, yielding a 7.45% response rate.

### Ethics Approval

The study protocol was reviewed and approved by the Hanyang University Institutional Review Board (HYIRB-202209-006).

### Questionnaires

The survey comprised 3 sections: demographics, the roles and functions of the community pharmacy during the pandemic, and the updated roles of the community pharmacy during disasters. The questions in the second and third sections were derived from the literature on pharmaceutical care, pharmacy practice, and the role of pharmacies during disasters. They were also modified to reflect the Korean context. The content of the developed questionnaire was pretested with members of the public, registered pharmacists, and researchers.

The demographic variables were sex, age, education, occupation, and region. For the statistical analyses, regions (8 *Si*s and 9 *Do*s) were recategorized into metropolitan, urban, and rural areas. Metropolitan areas included South Korea’s capital city, Seoul, and its surrounding areas, Incheon and Gyeonggi. Urban areas included the 6 *Si*s excluding Seoul and Incheon. Rural areas included the 8 *Do*s excluding Gyeonggi. Two additional items were added to the demographics: experience with COVID-19 and having a family pharmacy. In this study, *a family pharmacy* was defined as a pharmacy where a respondent would usually fill a prescription or ask for advice about his or her health problem [38-40]. The second section was composed of 28 questions regarding the roles and functions of community pharmacies during the pandemic. We presented 12 questions to assess the roles of community pharmacies. For the functions of community pharmacies, we categorized 16 questions into 4 domains: communication, knowledge, responsiveness, and collaboration. The third section includes 19 questions regarding the roles of community pharmacies during disasters. To clarify the updated roles of the community pharmacy during disasters, 4 disaster phases—prevention, preparedness, responses, and recovery—were used. Questions in the second and third sections were rated on a 5-point Likert scale from 1 (strongly disagree) to 5 (strongly agree). Detailed survey questions are provided in Multimedia Appendix 2.

### Statistical Analyses

Two types of analysis were applied: descriptive analysis and ordered logistic regression. Descriptive analysis was used to present the distribution of rated scores, mean scores, and SDs. A chi-square test compared categorical variables, including having a family pharmacy and experiencing COVID-19 [41]. Cronbach α was calculated to measure the internal consistency of survey items. An ordered logistic regression was conducted to elucidate the association between having a family pharmacy and positive perceptions toward community pharmacies. In the ordered logistic model, the dependent variable was ordinal scale data, and the independent variables were having a family pharmacy, experiencing COVID-19, sex, age, region, and education. We tested the proportional odds assumption using the Brant test, and if it was not satisfied, we conducted a generalized ordered logistic regression [42]. The results of the ordered logistic regression presented adjusted odds ratios (aORs) with 95% CIs. Statistical significance was considered at a 2-sided *P* value <.05. All statistical analyses were performed using Stata software (version 16; StataCorp).

## Results

### Survey Respondents

Table 1 presents the basic characteristics of the 1000 respondents to this study. Of the respondents, 418 (41.8%) had a history of COVID-19, and 639 (63.9%) had a family pharmacy. We separated the respondents into 2 groups: those with or without a family pharmacy. The distribution of respondents in the 2 groups was not significantly different in terms of sex, age, region, education, occupation, or COVID-19 infection (all *P*>.05).

**Table 1:** Characteristics of the survey respondents.

Characteristics			Total respondents (n=1000), n (%)		Respondents with a family pharmacy (n=639), n (%)		Respondents without a family pharmacy (n=361), n (%)		*P* value	
**Sex**										.39
	Male	496 (49.6)		324 (50.7)		172 (47.6)				
	Female	504 (50.4)		315 (49.3)		189 (52.4)				
**Age range (years)**										.07
	19-29	162 (16.2)		89 (13.9)		73 (20.2)				
	30-39	151 (15.1)		94 (14.7)		57 (15.8)				
	40-49	188 (18.8)		120 (18.8)		68 (18.8)				
	50-59	198 (19.8)		131 (20.5)		67 (18.6)				
	60 and older	301 (30.1)		205 (32.1)		96 (26.6)				
**Region**										.14
	Metropolitan	504 (50.4)		317 (49.6)		187 (51.8)				
	Urban	190 (19)		133 (20.8)		57 (15.8)				
	Rural	306 (30.6)		189 (29.6)		117 (32.4)				
**Education**										.36
	Below 12 years	221 (22.1)		135 (21.1)		86 (23.8)				
	13 years and above	779 (77.9)		504 (78.9)		275 (76.2)				
**Occupation**										.14
	White-collar worker	450 (45)		288 (45.1)		162 (44.9)				
	Blue-collar worker	141 (14.1)		91 (14.2)		50 (13.9)				
	Self-employed	90 (9)		62 (9.7)		28 (7.8)				
	Housewife	159 (15.9)		106 (16.6)		53 (14.7)				
	Student	25 (2.5)		10 (1.6)		15 (4.2)				
	Others	135 (13.5)		82 (12.8)		53 (14.7)				
**COVID-19 infection**										.65
	Yes	418 (41.8)		271 (42.4)		147 (40.7)				
	No	582 (58.2)		368 (57.6)		214 (59.3)				

### Assessment of the Roles of Community Pharmacies During the Pandemic

**Figure 1:**
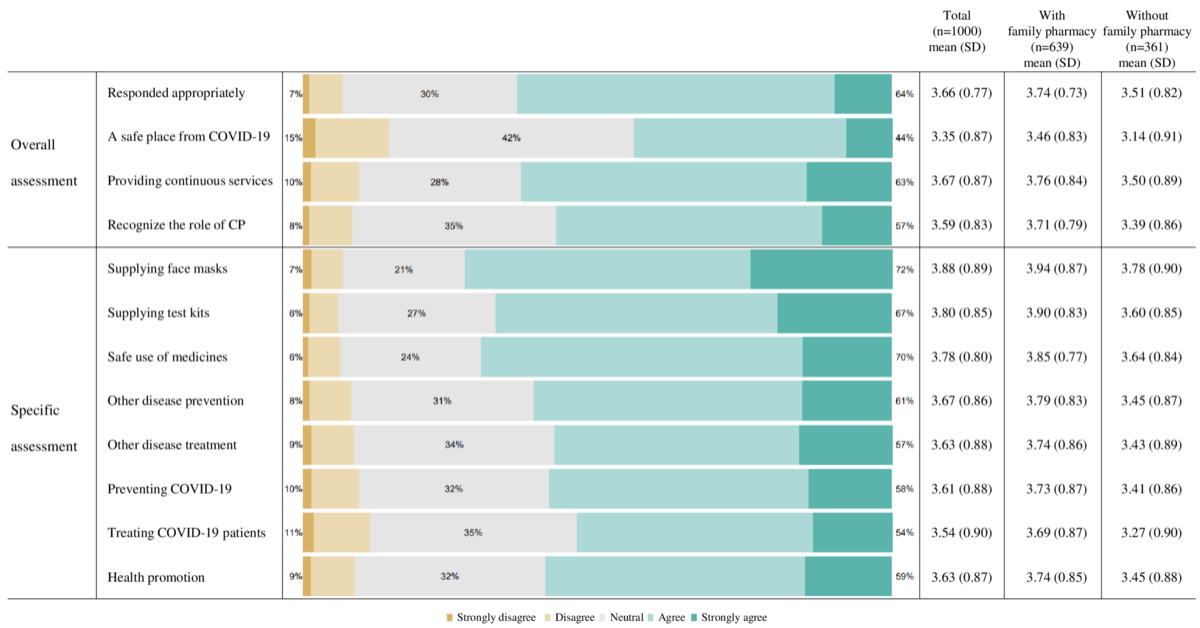
The assessment of the roles of community pharmacies (CP) during the pandemic.

Figure 1 describes the overall and specific assessments of the roles of community pharmacies during the pandemic. We reported the distribution of rated scores, mean scores, and SDs. A higher score indicates that the roles of community pharmacies are perceived as positive by the respondents. Regarding the overall assessment, respondents gave higher scores for community pharmacies that had responded appropriately (a mean Likert score of 3.66, SD 0.77 out of 5) and provided continuous pharmaceutical services (mean 3.67, SD 0.87). The pandemic served as an opportunity to positively recognize the roles of community pharmacies (mean 3.59, SD 0.83). However, respondents gave lower scores for community pharmacies as a safe place from COVID-19 (mean 3.35, SD 0.87). Respondents gave higher scores for community pharmacies playing an essential role in the supply of publicly distributed face masks (mean 3.88, SD 0.89) and COVID-19 rapid antigen test kits (mean 3.80, SD 0.85) and in the safe use of medicines (mean 3.78, SD 0.80). However, respondents gave lower scores for community pharmacies playing an essential role in treating patients with COVID-19 (mean 3.54, SD 0.90) and preventing COVID-19 (mean 3.61, SD 0.88). When we separated the respondents into 2 groups, higher scores were observed consistently in the subgroup of those with a family pharmacy than those without one.

### Perceived Functions of Community Pharmacies During the Pandemic

**Figure 2:**
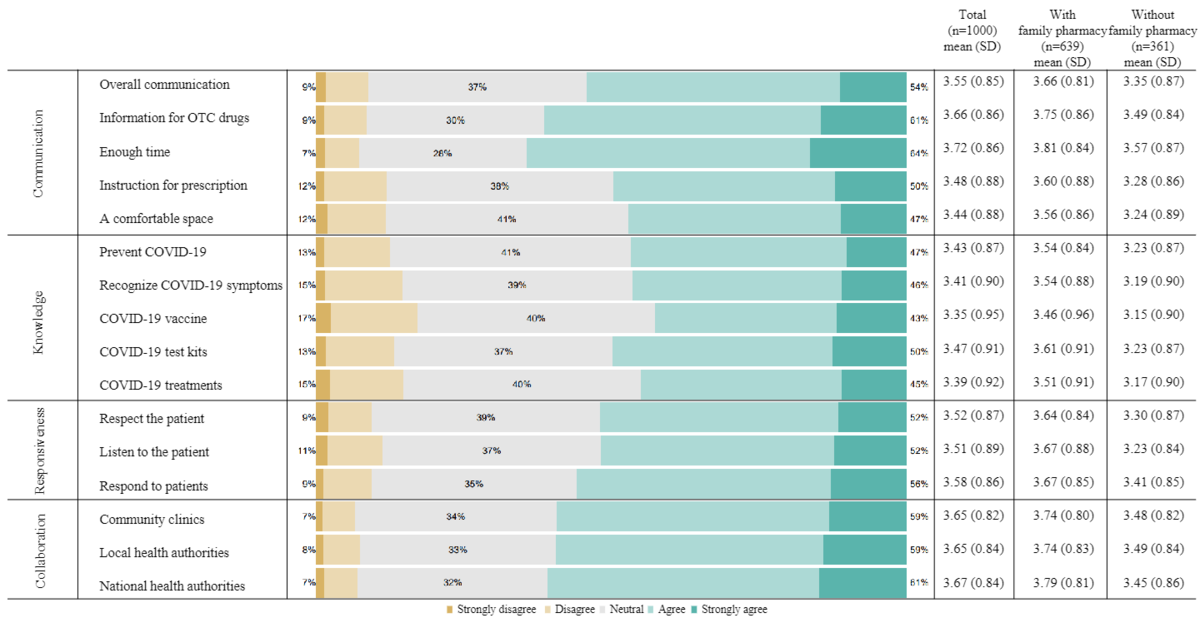
The perceived functions of community pharmacies during the pandemic. OTC: over-the-counter.

Figure 2 presents the perceived functions of community pharmacies during the pandemic. The mean score of the 4 domains was the greatest for collaboration (mean 3.66, SD 0.83), followed by communication (mean 3.57, SD 0.87), responsiveness (mean 3.54, SD 0.87), and knowledge (mean 3.41, SD 0.91). Respondents gave higher scores for community pharmacies collaborating with community clinics and hospitals (mean 3.65, SD 0.82), local health authorities (mean 3.65, SD 0.84), and national health authorities (mean 3.67, SD 0.84). However, respondents gave lower scores for the expertise of community pharmacies regarding the COVID-19 vaccine (mean 3.35, 0.95), COVID-19 treatment (mean 3.39, SD 0.92), and recognizing COVID-19 symptoms (mean 3.41, SD 0.90). Cronbach α values for the communication, knowledge, responsiveness, and collaboration domains were .88, .92, .83, and .85, respectively. When we separated the respondents into 2 groups, higher scores were observed consistently in the subgroup of those with a family pharmacy than those without one.

### Updated Roles of the Community Pharmacy During Disasters

**Figure 3:**
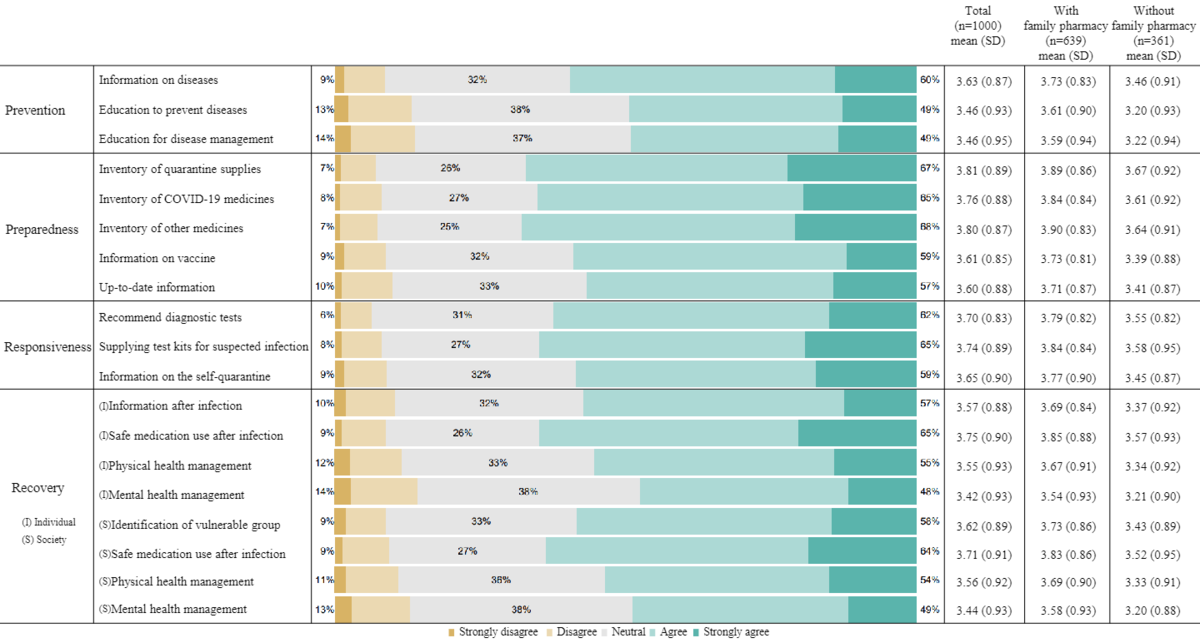
The updated roles of community pharmacies during disasters.

Figure 3 describes the updated roles of community pharmacies during disasters. The 4 phases in descending order according to mean scores were preparedness (mean 3.72, SD 0.88), responsiveness (mean 3.70, SD 0.87), recovery (mean 3.58, SD 0.92), and prevention (mean 3.52, SD 0.92). Respondents gave higher scores for the items belonging to the preparedness phase: an inventory of quarantine supplies (mean 3.81, SD 0.89), other medicines, including over-the-counter drugs (mean 3.80, SD 0.87), and medicines used for treating COVID-19 (mean 3.76, 0.88). Respondents gave higher scores for pharmacies supplying COVID-19 rapid antigen test kits (mean 3.74, SD 0.89) under the responsiveness phase and managing safe medication use after infection (mean 3.75, SD 0.90) under the recovery phase. However, respondents gave lower scores to pharmacies for providing education to prevent infectious disease (mean 3.46, SD 0.93) and education for infectious disease management (mean 3.46, 0.95) under the prevention phase. Cronbach α values for the prevention, preparedness, responsiveness, individual recovery, and social recovery domains were .86, .88, .81, .87, and .88, respectively. Higher scores were consistently observed in the subgroup of those with a family pharmacy than those without one.

### Having a Family Pharmacy and Positive Perceptions

Table 2 presents factors associated with a positive assessment of the roles of community pharmacies during the pandemic. Having a family pharmacy was consistently associated with a positive assessment of all items about the roles of community pharmacies during the pandemic. In particular, having a family pharmacy (aOR 1.67, 95% CI 1.23-2.15) and higher educational attainment (aOR 1.40, 95% CI 1.02-1.93) were associated with a positive assessment that community pharmacies responded appropriately during the pandemic.

**Table 2:** Ordered logistic regression analysis for each item in the assessment of the roles of community pharmacies (CP) during the pandemic.

Dependent variable^a^		Independent variable (reference), odds ratio (95% CI)						
		Family pharmacy: yes (no)	COVID-19 infection: yes (no)	Sex: female (male)	Age (years)	Education: ≥13 years (<13 years)	Region: metropolitan (rural)	Region: urban (rural)
**Overall assessment**								
	Responded appropriately^b^	1.669^c^ (1.298-2.147)	1.060 (0.831-1.352)	1.058 (0.830-1.348)	1.000 (0.991-1.010)	1.401^d^ (1.015-1.933)	1.277 (0.952-1.714)	0.766 (0.400-1.464)
	A safe place from COVID-19	1.970^c^ (1.531-2.536)	0.846 (0.667-1.071)	0.867 (0.684-1.100)	0.987^e^ (0.979-0.996)	1.366^d^ (1.010-1.847)	1.250 (0.894-1.746)	0.914 (0.701-1.191)
	Providing continuous services	1.790^c^ (1.396-2.295)	1.006 (0.796-1.270)	1.021 (0.805-1.295)	1.000 (0.991-1.008)	1.610^e^ (1.179-2.199)	1.105 (0.799-1.528)	1.099 (0.835-1.447)
	Recognize the role of CP	2.041^c^ (1.589-2.623)	1.087 (0.858-1.377)	0.824 (0.649-1.046)	1.001 (0.992-1.009)	1.323 (0.989-1.769)	0.932 (0.677-1.281)	0.889 (0.676-1.169)
**Specific assessment**								
	Supplying face masks	1.425^e^ (1.114-1.823)	0.825 (0.650-1.046)	1.136 (0.899-1.436)	1.001 (0.993-1.010)	1.272 (0.920-1.758)	0.928 (0.664-1.296)	0.846 (0.637-1.123)
	Supplying test kits	1.968^c^ (1.543-2.509)	0.935 (0.739-1.183)	0.940 (0.744-1.189)	0.995 (0.956-1.003)	1.141 (0.849-1.534)	1.198 (0.855-1.678)	0.886 (0.674-1.164)
	Safe use of medicines	1.676^c^ (1.282-2.114)	0.947 (0.743-1.207)	1.005 (0.788-1.281)	1.000 (0.991-1.009)	1.281 (0.934-1.758)	1.016 (0.724-1.424)	0.930 (0.702-1.233)
	Other disease prevention^b^	2.516^c^ (1.924-3.290)	0.942 (0.743-1.195)	0.978 (0.772-1.238)	1.006 (0.997-1.016)	1.195 (0.886-1.612)	1.302 (0.926-1.829)	1.138 (0.848-1.527)
	Other disease treatment	1.945^c^ (1.524-2.482)	0.989 (0.785-1.246)	0.997 (0.789-1.259)	0.997 (0.989-1.006)	1.345 (0.993-1.820)	0.971 (0.704-1.338)	1.027 (0.783-1.347)
	Preventing COVID-19	1.981^c^ (1.560-2.514)	0.886 (0.701-1.119)	0.961 (0.756-1.220)	1.000 (0.992-1.008)	1.362 (1.008-1.842)	1.097 (0.797-1.510)	0.759 (0.577-0.997)
	Treating COVID-19 patients	2.460^c^ (1.926-3.143)	1.049 (0.832-1.322)	0.987 (0.780-1.249)	0.991^d^ (0.983-1.000)	1.131 (0.831-1.540)	1.059 (0.761-1.474)	0.981 (0.743-1.294)
	Health promotion^b^	1.795^c^ (1.407-2.290)	1.057 (0.836-1.337)	0.859 (0.679-1.086)	1.008 (0.999-1.018)	1.401^d^ (1.046-1.876)	1.420 (0.977-2.063)	1.271 (0.947-1.705)

^a^Value of dependent variable: 1=Strongly disagree, 2=Disagree, 3=Neutral, 4=Agree, and 5=Strongly agree.

^b^Generalized ordered logistic regression (1, 2, and 3 vs 4 and 5).

^c^*P*<.001.

^d^*P*<.05.

^e^*P*<.01.

## Discussion

### Study Overview

This study assessed the perceived roles and functions of community pharmacies during the pandemic and explored their updated roles after the pandemic. The expanded roles of community pharmacies requested by the community accelerated quickly during the pandemic. Furthermore, community pharmacies began to develop new functions to address the pandemic. Findings from this study highlight how to expand and update the roles and functions of community pharmacies in countries where pharmacies are still limited to medicine-centered services.

### Principal Findings in Pharmacy Practice

Three findings are noteworthy. First, in the overall assessment, more than 50% of participants agreed that community pharmacies responded appropriately, and the pandemic served as an opportunity to recognize the role of community pharmacies. When we asked for specific assessments, the proportion of participants who agreed was the highest for the question that pharmacies that had played an essential role in supplying face masks, followed by COVID-19 test kits and advice about the safe use of medicines. Assigning specific roles and functions to community pharmacies would contribute to the public’s positive assessment of community pharmacies. Similarly, community pharmacies in other countries have shown effort and courage in providing face masks and COVID-19 test kits and administering vaccinations [6,43,44]. The public assessed effort and courage positively.

Second, we conducted this study as part of a more extensive study on the public perceptions of community pharmacies in South Korea. By comparing the previous and current studies, we could evaluate the changed roles and functions of community pharmacies before and during the pandemic. As explained earlier, these 2 studies implemented a quota sampling method stratified by age, sex, and region. Both studies conducted a self-reported web-based survey for 1000 adults aged older than 19 years. Furthermore, the questionnaires in the 2 studies were closely related. We provided 4 domains to understand the functions of community pharmacies. Changes in the public perceptions toward the functions of community pharmacies were noted. In this study, the mean Likert scores of the 4 domains in descending order were collaboration (3.66), communication (3.57), responsiveness (3.54), and knowledge (3.41). In contrast, the mean Likert scores of the 4 domains in descending order were knowledge (3.73), responsiveness (3.47), communication (3.45), and collaboration (3.44) in the previous study [11]. The mean Likert score for collaboration, communication, and responsiveness increased by 0.22, 0.12, and 0.07 points, respectively, whereas the score for knowledge decreased by 0.32 points. Knowledge has been essential to interprofessional collaboration between community pharmacists and general practitioners [45,46]. However, community pharmacists still need to develop their knowledge, particularly about COVID-19, during the pandemic. In contrast, the public responded that community pharmacies collaborated with community clinics and health authorities during the pandemic. The expertise of community pharmacists is required to build solid interprofessional collaboration between community pharmacists and general practitioners after the pandemic.

Third, we asked about the updated roles of community pharmacies in 4 disaster phases—prevention, preparedness, responsiveness, and recovery. Respondents gave higher scores for the items in preparedness and responsiveness, whereas they gave lower scores for the items in prevention and recovery. The preparedness and responsiveness phases were key areas during the pandemic [47]. However, community pharmacies’ role in prevention and recovery should not be underestimated [48,49]. The role of community pharmacies in the infection prevention phase is to take measures to reduce the health risk of disasters, including providing information and education about infections and ensuring that patients are aware of the increased risk of adverse health outcomes [24,32]. The role of community pharmacies in the recovery phase involved checking on the local community’s health needs, identifying and prioritizing susceptible patients, and participating in postdisaster reports [32,47]. In European countries, a comprehensive range of pharmacy practices, from prevention to recovery, has been implemented to address the demands during a pandemic [24]. By doing so, community pharmacies in other countries could be a bridge between medical care and broader community services. Furthermore, community pharmacies could be embedded in the comprehensive case management of patients with COVID-19 and other susceptible groups [50,51]. In this study, having a family pharmacy was consistently associated with positive assessments by the public. The consistent effect implies that family pharmacies could be a platform for embedding community pharmacies into the comprehensive case management of patients.

### Policy Implications: Barriers and Facilitators in Expanding the Roles of Community Pharmacies

It is necessary to review the mean score of each survey item. The scores ranged from 3.35 to 3.88 points, implying that they were less than “agree” or “positive.” The government and researchers in South Korea praised the roles of community pharmacies in distributing face masks in the early phase of the pandemic [52-54]. However, in the survey, respondents gave ratings of less than “agree (4.00 pts)” when asked if community pharmacies had played an essential role in the supply of publicly distributed face masks. Which factors caused the difference in assessment between the public and the government and researchers? In addition, what caused the public to perceive the roles of community pharmacies during the pandemic as less than positive?

First, we should note the timing factor. We conducted this survey in October 2022, and community pharmacies supplied face masks from March 2020 to July 2020. A more than 2-year gap between the 2 events could have caused the public’s neutral stance. The lack of expertise is another factor. Similar to retailers, community pharmacies supplied face masks to the public. Pharmacies, post offices, and Nonghyup Hanaro Mart—a grocery store—supplied face masks during the initial stage of the pandemic. However, the public did not expect the distribution of face masks to be a substantial role of community pharmacies. The public also responded that community pharmacies needed to gain knowledge. In the questionnaire, the public gave the lowest score to community pharmacists who provided expertise on the COVID-19 vaccine. Community pharmacists in Egypt expressed an unmet need for pandemic-related knowledge as a barrier to the expanded roles of community pharmacies [44]. The lack of expertise requires measures such as providing continuous guidance on the pandemic and educational materials to community pharmacists. Space is the final factor. Our previous research raised time and space issues in the communication between pharmacists and patients [11]. Before the pandemic, we noted the availability of private and comfortable spaces within community pharmacies as barriers to expanding the roles of community pharmacies. During the pandemic, the public raised space issues regarding coronavirus transmission within the pharmacy and responded that community pharmacies were unsafe from COVID-19. Improper infection control measures in community pharmacies, reported in Australia, provided clues to interpret this finding [55]. In South Korea, approximately 60% of community pharmacies had a total space of less than 66 m^2^, implying that a separate space for infection control was impossible in most cases [56].

This study also highlights the association between community pharmacies’ roles and functions and pharmacists’ expertise. Pharmacists in South Korea do not have the authority to direct refill reviews and approvals and administer vaccinations [11]. During the pandemic, the roles of community pharmacists were limited to supplying face masks and COVID-19 test kits. Their roles did not expand to more active and professional ones, such as vaccinating the public and finding, preventing, and treating COVID-19 cases. In this context, the public might not expect the expertise of community pharmacists in areas they have yet to experience. Similarly, community pharmacists have not been incentivized to develop their expertise in those areas [57]. Assigning new roles to community pharmacies would contribute to developing the expertise of community pharmacists. Similarly, developing the expertise of community pharmacists would successfully link to expanding the roles and functions of community pharmacies.

### Strengths and Limitations

This study has several strengths. First, we analyzed public perceptions toward the roles and functions of community pharmacies. Many studies have captured the roles and functions of community pharmacies from the perspective of health care providers. However, public perceptions are essential in designing patient-centered community pharmacies. Second, we conducted a large-scale (n=1000) web-based questionnaire that represented the entire population in South Korea in terms of sex, age, and region. Third, we conducted this study as part of a more extensive study on the public perceptions of the roles and functions of community pharmacies. Before the COVID-19 pandemic, we studied the public perceptions of the roles and functions of community pharmacies in South Korea. This study analyzed the public perceptions of the roles and functions of community pharmacies during the COVID-19 pandemic. By comparing the different study results between the prepandemic and pandemic periods, we could investigate the effect of the pandemic on the public perceptions of the roles and functions of community pharmacies.

This study has several limitations. First and foremost, this study used a web-based survey and applied a stratified sampling method to obtain samples that represented the entire population in South Korea. The response rate was low for some quotas, and the total response rate was 7.45%. A low response rate implies that the study participants are a self-selected minority, and their participation might be linked to other personal characteristics. There would be selection bias in the sampling process, and the conclusion would be biased. Second, we investigated public perceptions to assess the roles and functions of community pharmacies during the pandemic and to explore the updated roles of community pharmacies after the pandemic began. However, measuring perceptions is problematic when allowing respondents to rate different items on the questionnaire. Third, this study was a cross-sectional survey, which may hinder the interpretation of the findings as a causal relationship. Longitudinal design and qualitative studies are needed to understand the contexts and mechanisms of public perceptions.

### Conclusions

The COVID-19 pandemic has changed the roles and functions of community pharmacies. Assigning specific roles and functions to community pharmacies contributed to the public’s positive assessment. Furthermore, having a family pharmacy was consistently associated with the public’s positive assessments. The pandemic resulted in interprofessional collaboration between community pharmacists and general practitioners. In particular, family pharmacies could be a valuable platform in which to embed community pharmacies in the comprehensive case management of patients. However, community pharmacists should have the expertise to build solid interprofessional collaborations and fulfill their expanded and updated roles.

## Appendix

Multimedia Appendix 1 The characteristics of the previous and current studies.

Multimedia Appendix 2 Detailed survey questions.

## Abbreviations

aOR: adjusted odds ratio
