# Supplementary material for: Impact of the COVID-19 Pandemic on the Public Perceptions of the Roles and Functions of Community Pharmacies in South Korea: Updated Cross-Sectional Self-Reported Web-Based Survey
Source: JMIR Public Health Surveill. 2023 Jul 13;9:e46723. doi: 10.2196/46723 (PMC10453941; doi:10.2196/46723)
Supplement: Multimedia Appendix 1 [file publichealth_v9i1e46723_app1.pptx]

## Slide 1
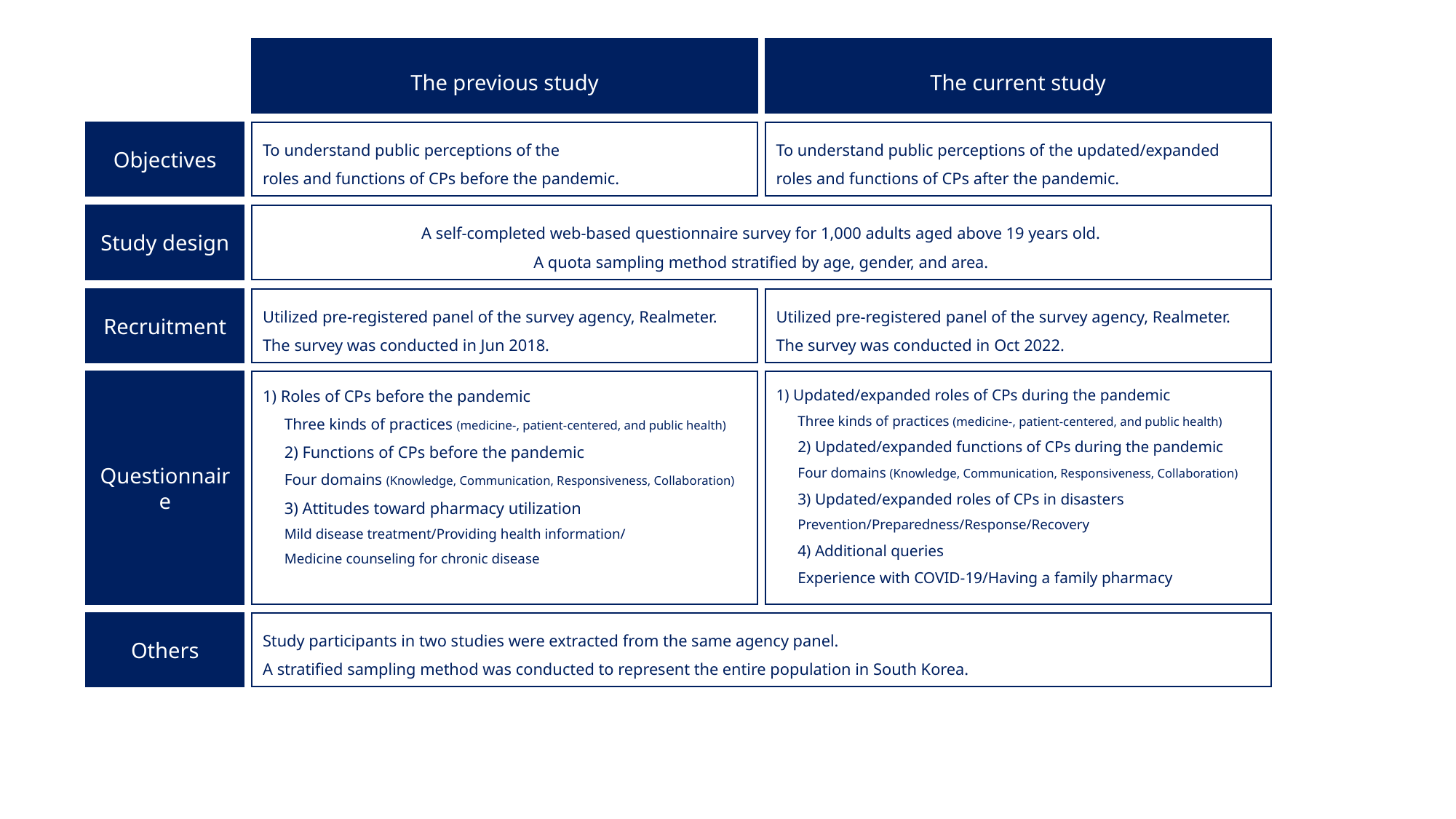

The previous study
The current study
Objectives
To understand public perceptions of the
roles and functions of CPs before the pandemic.
To understand public perceptions of the updated/expanded roles and functions of CPs after the pandemic.
Study design
A self-completed web-based questionnaire survey for 1,000 adults aged above 19 years old.
A quota sampling method stratified by age, gender, and area.
Recruitment
Utilized pre-registered panel of the survey agency, Realmeter.
The survey was conducted in Jun 2018.
Utilized pre-registered panel of the survey agency, Realmeter.
The survey was conducted in Oct 2022.
Questionnaire
1) Roles of CPs before the pandemic
Three kinds of practices (medicine-, patient-centered, and public health)
2) Functions of CPs before the pandemic
Four domains (Knowledge, Communication, Responsiveness, Collaboration)
3) Attitudes toward pharmacy utilization
Mild disease treatment/Providing health information/
Medicine counseling for chronic disease
1) Updated/expanded roles of CPs during the pandemic
Three kinds of practices (medicine-, patient-centered, and public health)
2) Updated/expanded functions of CPs during the pandemic
Four domains (Knowledge, Communication, Responsiveness, Collaboration)
3) Updated/expanded roles of CPs in disasters
Prevention/Preparedness/Response/Recovery
4) Additional queries
Experience with COVID-19/Having a family pharmacy
Others
Study participants in two studies were extracted from the same agency panel.
A stratified sampling method was conducted to represent the entire population in South Korea.
