# Supplementary material for: Impact of the COVID-19 Pandemic on the Public Perceptions of the Roles and Functions of Community Pharmacies in South Korea: Updated Cross-Sectional Self-Reported Web-Based Survey
Source: JMIR Public Health Surveill. 2023 Jul 13;9:e46723. doi: 10.2196/46723 (PMC10453941; doi:10.2196/46723)
Supplement: Multimedia Appendix 2 [file publichealth_v9i1e46723_app2.docx]

**Detailed Survey Questions**

1. Roles and functions of the community pharmacy during the pandemic
2. Roles of the community pharmacy
3. Overall assessment (4 items)

: Community pharmacies responded appropriately during the pandemic

: Community pharmacies were a safe place from COVID-19

: Community pharmacies provide continuous pharmaceutical services

: The pandemic served as an opportunity to recognize the role of community pharmacies positively

1. Specific assessment (8 items)

: Community pharmacies played an essential role in the supply of publicly distributed face masks early in the pandemic

: Community pharmacies played an essential role in the supply of COVID-19 rapid antigen test kits

: Community pharmacies play an essential role in the safe use of medicines

: Community pharmacies play an important role in disease prevention (non-COVID-19 diseases)

: Community pharmacies play an essential role in the treatment of diseases (non-COVID-19 diseases)

: Community pharmacies played an essential role in preventing COVID-19

: Community pharmacies played an essential role in treating COVID-19 patients

: Community pharmacies play an important role in overall health promotion

1. Functions of the community pharmacies
2. Communication (5 items)

: I was satisfied with my communication with pharmacists during the pandemic

: Community pharmacists provided the necessary information for over-the-counter drugs during the pandemic

: Community pharmacists provided necessary instructions for prescription medications during the pandemic

: Community pharmacists spend enough time with patients during the pandemic

: Community pharmacies provided a comfortable space for communication during the pandemic

1. Knowledge (5 items)

: Community pharmacists provided expertise to prevent COVID-19 during the pandemic

: Community pharmacists provided expertise to recognize COVID-19 symptoms during the pandemic

: Community pharmacists provided expertise in the COVID-19 vaccine during the pandemic

: Community pharmacists provided expertise in COVID-19 rapid antigen test kits during the pandemic

: Community pharmacists provided expertise to treat COVID-19 during the pandemic

1. Responsiveness (3 items)

: Community pharmacists respected me during the pandemic

: Community pharmacists listened to my needs carefully during the pandemic

: Community pharmacists responded appropriately to my needs during the pandemic

1. Collaboration (3 items)

: Community pharmacists collaborated with community clinics and hospitals

: Community pharmacists collaborated with local health authorities (e.g., public health centers)

: Community pharmacists collaborated with national health authorities (e.g., the Ministry of Health and Welfare)

1. Updated roles of the community pharmacies
2. Prevention (3 items)

: Providing information on infectious diseases and symptoms

: Providing education to prevent infectious diseases

: Providing education for infectious disease management

1. Preparedness (5 items)

: Having an appropriate inventory of quarantine supplies, including hand sanitizers, face masks, rapid antigen test kits

: Having an appropriate inventory of medicines used for treating COVID-19

: Having an appropriate stock of other medicines and over-the-counter drugs

: Providing information related to the COVID-19 vaccine

: Providing up-to-date information on infectious diseases

1. Responsiveness (3 items)

: Recommending diagnostic tests and treatment for patients with suspected infection

: Supplying COVID-19 rapid antigen test kits for patients with suspected infection (including education on how to use the kits)

: Providing information on symptoms and treatment during the self-quarantine

1. Recovery (8 items)
2. Individual level

: Providing information on the after-effects of the infection

: Management for safe medication use after the infection

: Physical health management after the infection

: Mental health management after the infection

1. Society level

: Identification of the vulnerable groups requiring pharmaceutical care

: Management for safe medication use for vulnerable groups

: Physical health management for the vulnerable groups

: Mental health management for the vulnerable groups
